# Primary signet-ring cell carcinoma of the urinary bladder

**DOI:** 10.4322/acr.2021.264

**Published:** 2021-04-26

**Authors:** Nirmalya Benerjee, Kalpesh Parmar, Kim Vaiphei

**Affiliations:** 1 Post Graduate Institute of Medical Education and Research, Department of Pathology, Chandigarh, India; 2 Post Graduate Institute of Medical Education and Research, Department of Urology, Chandigarh, India

**Keywords:** Cystectomy, Carcinoma, Signet-Ring Cell, Urinary Bladder

## Abstract

Primary signet-ring cell carcinoma of the urinary bladder is a rare tumor. The overall incidence is approximately 0.12-0.6% of all urinary bladder malignancies. The majority of the patients present in an advanced stage with a uniformly grim prognosis. As signet-ring cell carcinomas are more common in the gastrointestinal tract, a possibility of metastasis needs to be considered. Here we report, a 42-year-old patient who presented with hematuria and was diagnosed with a urinary bladder tumor. The patient was managed with partial cystectomy and pelvic lymph node dissection. The histopathological examination confirmed primary signet-ring cell carcinoma of the urinary bladder.

## INTRODUCTION

Primary signet-ring cell carcinoma (PSRCC) of the urinary bladder is a rare type of adenocarcinoma and was first reported in 1955 by Saphir et al.^1^ The overall prevalence remains 0.12-0.6% of all primary malignancies of the urinary bladder.^2^ The majority of patients are in their seventh to the eighth decade of life with male preponderance. Approximately 25% of patients show distant metastasis, and about 50% of cases present with locally advanced disease at the time of diagnosis.^3^ PSRCC arises from the urothelium. Urothelium undergoes glandular metaplasia secondary to chronic irritation and is thought to be a precursor lesion of PSRCC. Clinical presentation is similar to other primary bladder tumors, which is hematuria. Another rare presentation is mucinuria reported in 3-12% of the individuals.^4^ Radiological features are often non-specific. Therefore, cystoscopic evaluation is extremely important. However, in the linitis-plastica variant, no mucosal lesion is seen on cystoscopy. Hence early diagnosis becomes difficult in these cases.^5^ Signet-ring cell carcinoma is far more common outside the urinary bladder and is mostly seen in the gastrointestinal tract. Hence the possibility of metastasis should always be considered, and a thorough workup is needed to rule out primary elsewhere.^6^ ^,^ ^7^ Surgical therapy remains the mainstay of treatment with or without postoperative chemotherapy. The prognosis remains uniformly poor in PSRCC. Elevated serum CEA level and diffuse linitis plastica like morphology are usually associated with a grim prognosis.^8^ We present a case of primary signet-ring cell carcinoma located in the urinary bladder’s dome in a 42-year-old male presenting with hematuria who was managed with partial cystectomy and pelvic lymph node dissection. The diagnostic approach, differentiating features, prognosis, and survival have been discussed.

## CASE REPORT

**Figure 1:**
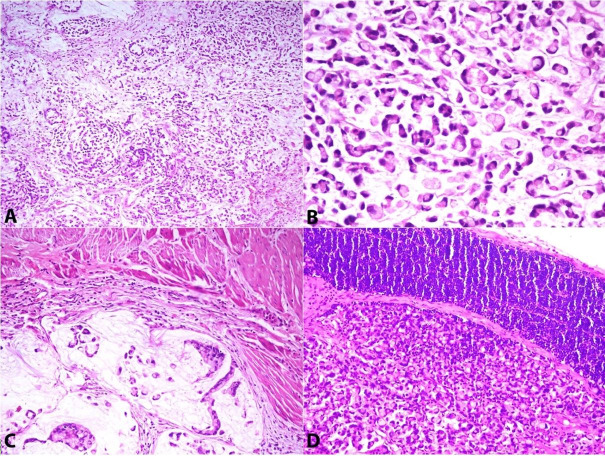
Photomicrography of tumor. **A –** Signet-ring cells are diffusely scattered with extracellular mucinous lakes (H&E, 4X); **B –** Signet-ring cells with intracellular mucin and nuclei pushed to the periphery (H&E, 40X); **C –** Tumor cells are infiltrating muscularis propria (H&E, 20X); **D –** Tumor cells have metastasized to the lymph node (H&E, 20X).

A 42-year-old male presented to the urology outpatient clinic with painless and gross hematuria with clots of four weeks duration. He was a chronic smoker but denied loss of weight or appetite. His past medical and family history was non-remarkable. On examination, the patient was conscious, alert and his vitals were stable. Systemic examination was grossly normal. Digital rectal examination showed grade 1 prostate, benign, and the lateral pelvic wall appeared normal. The abdominal ultrasound reported a solitary 3x2 cm papillary growth in the dome of the urinary bladder. The rest of the urinary bladder and bilateral kidneys were normal. His routine laboratory workup was within normal limits. Urine cytology revealed abundant atypical cells. The patient was counseled and planned for transurethral resection of the urinary bladder tumor (TURBT) under regional anesthesia. Using a 26-French resectoscope and 30-degree lens, a complete resection of the tumor was done, and a deep muscle biopsy was sent separately for assessment. The postoperative course was uneventful. The histopathological examination showed an invasive urothelial tumor with glandular differentiation suggestive of adenocarcinoma. The detrusor muscle invasion was present. At 3 weeks of follow up, a staging workup was done. The abdominal contrast-enhanced computed tomography (CECT) revealed mild thickening in the dome of the urinary bladder and a perivesical fat stranding with an enlarged right obturator lymph node measuring 16x7 mm. The positron emission tomography (PET) scan revealed fluorodeoxyglucose (FDG) avid uptake in the dome of the urinary bladder and right obturator node with no other focus elsewhere (standardized uptake values max 6.1). The chest x-ray, liver function test, upper gastrointestinal endoscopy, and colonoscopy were unremarkable. Serum prostate-specific antigen (PSA) was of 2.1 ng/ml (reference range [RR]; 0-4 ng/ml), CA 19-9 of 10 IU/ml (RR: 0-37 U/ml) and carcinoembryonic antigen (CEA) of 1.9 ng/ml (RR: < 2.5 ng/ml). In view of the localized disease to the dome of the urinary bladder and histopathology reporting as adenocarcinoma, partial cystectomy with bilateral pelvic lymph node dissection was planned. A midline infra umbilical laparotomy was undertaken. The urinary bladder’s dome was excised with a 2 cm margin all around the scar of previous surgery, along with a standard bilateral pelvic lymph node resection was performed. The post-operative was uneventful. The surgical specimen consisted of an unoriented partial cystectomy measuring 40x26x20 mm. A solitary exophytic growth was noted in the mucosa measuring 20x20x8mm. Multiple sections were taken, including the soft tissue resection limits. Sections study showed tumor cells’ sheets with an occasional glandular configuration in a background of abundant extracellular mucin (Figure 1A). The tumor cells were predominantly (>90%) signet-ring-shaped with intracellular mucin accumulation (Figure 1B). The tumor cells infiltrated up to the inner half of the superficial muscularis propria (Figure 1C). No extension into the perivesical tissue was noted. Lymphatic emboli were present, but no perineural invasion. The tumor was 7 mm away from the surgical soft tissue resection planes. Only 2 of the 5 lymph nodes of the right pelvic group and none of the 6 lymph nodes of the left pelvic group showed metastasis (Figure 1D).

The final histopathological impression was T2aN2M0 Signet-ring cell carcinoma. A panel of immunostainings (Table 1) was carried out to differentiate between a primary and metastatic tumor. Diffuse positivity for CEA and CK20 were detected (Figure 22B). Nuclear positivity for CDX2 was detected, and E- cadherin was retained by the tumor cells (Figure 22D).

**Table 1:** Results of IHC

Positive reaction	CEA	CK20 (m)	CDX2 (n)	E-cadherin (m)
Negative reaction	GATA3	PSA	CD138	β-catenin

M= membrane; n= nuclear.

**Figure 2:**
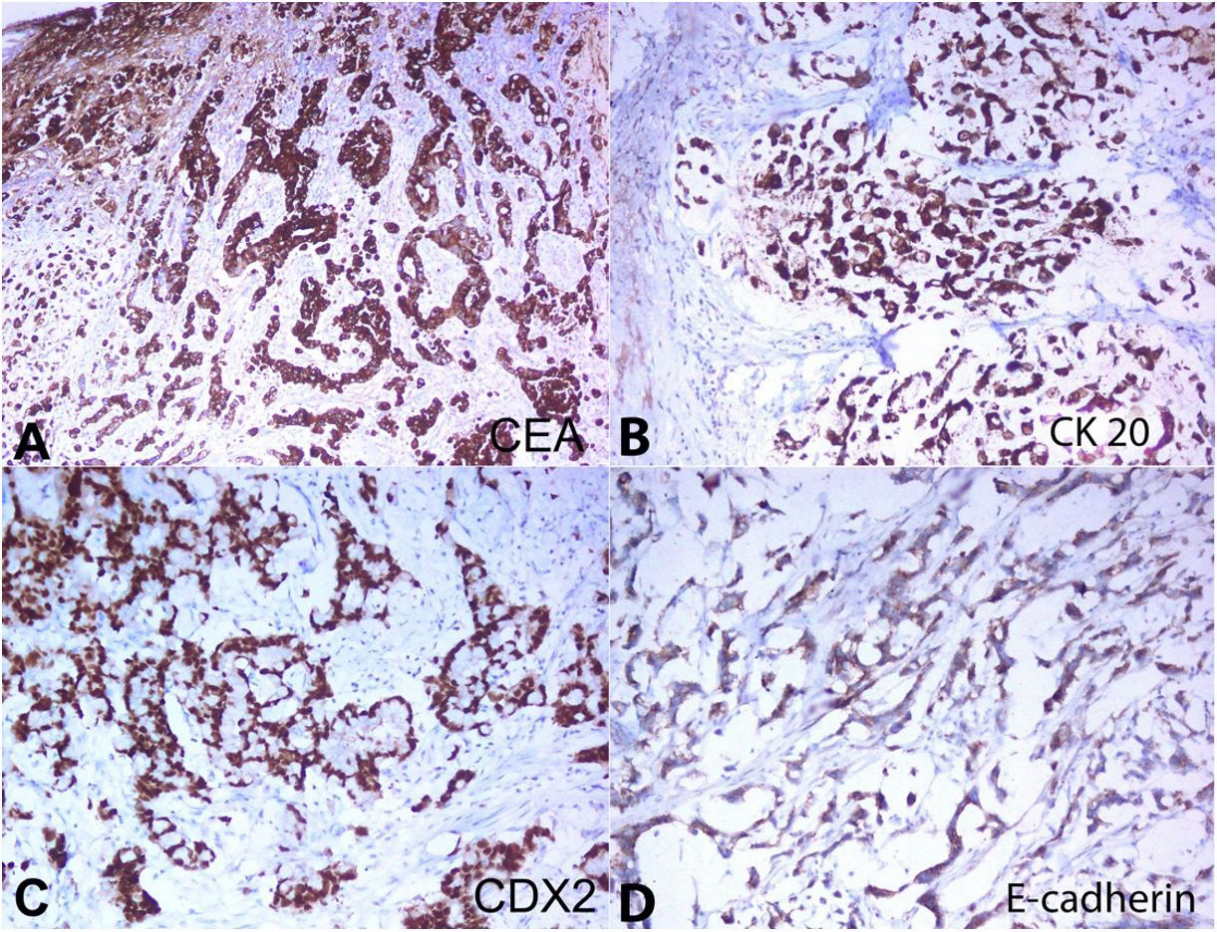
Photomicrography of bladder tumor. **A –** Tumor cells are diffusely positive for CEA (10X); and **B –** CK20 (10X); **C –** Nuclear positivity for CDX2 is noted (20X); **D –** Membranous positivity for E-cadherin is seen (20X).

**Figure 3:**
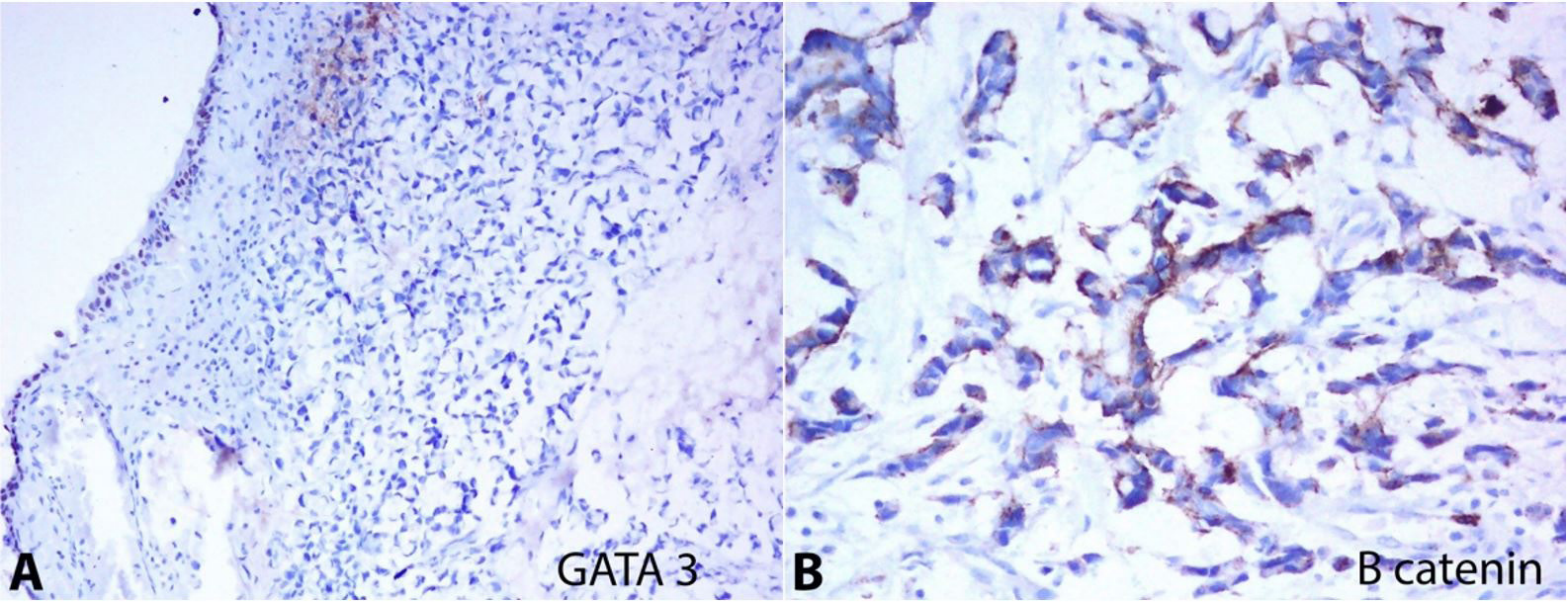
Photomicrography of the bladder tumor. **A –** Tumor cells are negative for GATA3; internal control positive (10X); **B –** β- catenin immunostaining shows the absence of nuclear positivity (20X).

No positivity was observed for PSA, GATA3 (Figure 3A), and AMACR. Faint and patchy positivity for CK7 was noted. Immunostain for β-catenin (Figure 3B) showed no nuclear staining, and CD138 immunostaining failed to stain any of the tumor cells. Based on these immune profiles, it confirmed primary signet-ring cell carcinoma of the urinary bladder.

The per-urethral catheter was removed after 3 weeks. The patient was referred to the Medical oncology unit in view of locally advanced disease, and he received 4 cycles of double-dense methotrexate, vinblastine, doxorubicin, and cisplatin regimen. The patient tolerated the chemotherapy regimen well. At 6 months follow-up, a check cystoscopy was normal, and the CECT of the chest and abdomen showed no evidence of recurrence. The patient was planned for cystoscopy surveillance at 3 monthly intervals for the next 2 years.

## DISCUSSION

PSRCC was first described in 1955. About 300 cases have been published, so far, in the English literature.^2^ The prognosis of PSRCC remains uniformly poor as it presents in an advanced stage and is commonly resistant to chemo and radiotherapy.^1^ ^,^ ^2^ This tumor commonly affects elderly male patients mostly in their sixth decades and characterizes a rapidly aggravating clinical course.^6^ Approximately 65% of the patients present with hematuria.^7^ On imaging, it may appear as diffuse wall thickening of the urinary bladder. At cystoscopy, and two-third of the cases present with exophytic growth. However, even urothelial carcinoma may present with diffuse fibrous thickening of the wall without any obvious mucosal lesion, which challenges the cystoscopic detection.^8^ ^-^ ^10^ Reactive proliferation is sometimes seen in the native urothelium (von-Brunn nest), mostly secondary to inflammation or chronic irritation, which is also responsible for cystic changes and subsequent glandular or intestinal metaplasia of the urothelium. This metaplastic mucosa acts as a precursor lesion of invasive adenocarcinomas, including signet ring cell carcinomas.^11^ Three, theories have been proposed in the published literature regarding the pathogenesis of PSRCC of the urinary bladder, namely (i) the PSRCC arises from the metaplastic epithelium of the cystitis cystica, and (ii) the neoplastic transformation of the totipotent urothelial cells without any metaplastic changes. Lastly, the urinary bladder PSRCC can also arise from the isolated signet-ring cell from the urothelium.^7^ ^,^ ^11^ ^,^ ^12^

The cytomorphological detail of signet ring cell carcinoma has been described by Guan et al.^13^ According to them, the cytospin smears were hypercellular with mixed inflammatory cells in the background. Scattered signet ring cells were described as large cells with eccentrically placed nuclei, prominent nucleoli, and discrete mucin vacuoles in the abundant cytoplasm. In primary urothelial carcinoma with signet ring cell features, the signet ring cells were admixed with coexisting malignant glandular epithelial cells.^13^ The histomorphological features of urinary bladder PSRCC is quite characteristic. Loosely cohesive signet-ring cells are scattered containing intracellular mucin abutting nuclei to the periphery.^2^ While diagnosing urinary bladder PSRCC, it is mandatory to rule out metastatic tumors from the gastrointestinal tract. The immunostainings for CK7, CK20, CEA, EMA, and CDX2 cannot differentiate the urinary bladder PSRCC from the primary signet-ring cell carcinoma of the gastrointestinal tract as the immunohistochemical profile often overlaps. However, the absence of nuclear positivity for β-catenin with no endoscopically detectable lesion in the gastrointestinal tract favors urinary bladder origin.^14^ In the present case, the tumor cells were positive for CEA, CK20, and CDX2. β-catenin positivity was restricted to the cell membrane, supporting the primary bladder origin. It is essential to mention that gathering information on immunohistochemistry, clinical features, imaging, and other invasive diagnostic modalities plays a vital role in reaching a definite diagnosis.^2^ Hence, upper and lower gastrointestinal endoscopy and chest X-ray have been done in this case to rule out the possibility of other origin sites. Another crucial differential diagnosis of PSRCC is the plasmacytoid variant of urothelial carcinoma. The plasmacytoid variant of urothelial carcinoma is a rare and aggressive variant showing plasmacytoid differentiation with signet-ring cells, and the cells are positive for CK7, GATA3, CD138, and CK20. Loss of E-cadherin is also an important additional finding present in the plasmacytoid variant of urothelial carcinoma, which was not seen in the index case.^14^ ^,^ ^15^ The possibility of primary urachal adenocarcinoma was also thought as the location of the tumor was in the dome of the bladder. Almost all urachal neoplasms arise from the dome. Amin et al.^16^ conducted one of the larger studies on the urachal tumors. They included a total of 55 cases, and out of them, 24 cases (44%) were invasive non-cystic adenocarcinoma. Out of these 24 cases of adenocarcinoma, five cases show signet ring cells in isolated small clusters and single scattered. Only one case showed diffusely infiltrative signet ring cells with 80% cells of signet ring cell morphology.^16^ There are very few published case reports of urachal signet ring cell carcinoma. Therefore, primary urachal adenocarcinoma with isolated signet-ring cell differentiation is very rare and was not considered in this case.^17^ ^,^ ^18^

As there is no consensus guideline for the management of PSRCC, input from the diagnostic and related clinical subspecialties are required in a multidisciplinary setting. PSRCCs are uniformly insensitive to radiotherapy and standard chemotherapy. Therefore, surgery remains the mainstay of treatment in 70% of the cases.^3^ ^,^ ^19^ Theoretically, surgeons can avail of transurethral resection to radical cystectomy with urinary diversion. However, partial cystectomy is generally preferred over radical cystectomy, especially in small, well-demarcated, localized tumors located in the urinary bladder’s dome.^2^ ^,^ ^20^ When a cisplatin-based combined chemotherapy regimen follows surgical therapy, it improves the progression-free and overall survival.^21^ The index case underwent partial cystectomy with standard pelvic lymph node dissection. In follow up, the patient received 3 cycles of adjuvant chemotherapy and is currently disease-free on follow up.

## CONCLUSION

Primary signet-ring cell carcinomas of the urinary bladder are uncommon. One should rule out focus at other primary landing sites. Staging workup is essential in all cases to rule out metastasis. Histopathological examination and immunostaining is key to diagnosis. Though highly aggressive, partial cystectomy is a feasible option in some cases. Follow up is life-long to look for recurrence of the disease.
